# Supplementary material for: The abscopal effect of IRE combined with anti–PD-1 achieves local ablation and systemic control of PDAC
Source: bioRxiv. 2026 May 7:2026.05.04.722535. Preprint. [Version 1] doi: 10.64898/2026.05.04.722535 (PMC13174494; doi:10.64898/2026.05.04.722535)
Supplement: Supplement 1 [file media-1.pdf]

The abscopal effect of IRE combined with anti–PD-1 achieves local ablation and systemic control of PDAC

Qizhen Cao et al.

Supplemental Information

Table S1. Top 21 upregulated DEGs at 4 h after IRE identified by RNAseq analysis.*

Rank	10p IRE	Encoding protein	20p IRE	Encoding protein
1	*Mmp25*	matrix metallopeptidase 25	*Mmp25*	matrix metallopeptidase 25
2	*Npas4*	neuronal PAS domain protein 4	*Npas4*	neuronal PAS domain protein 4
3	*Pecam1*	platelet and endothelial cell adhesion molecule 1 (CD31)	*Pecam1*	platelet and endothelial cell adhesion molecule 1 (CD31)
4	*Fam71a*	family with sequence similarity 71, member A	*Aldh3b2*	aldehyde dehydrogenase 3 family member B2
5	*Aldh3b2*	aldehyde dehydrogenase 3 family member B2	*Efr3b*	EFR3 homolog B
6	*Ampd1*	adenosine monophosphate deaminase 1	*Fam71a*	family with sequence similarity 71, member A
7	*Itgam*	integrin subunit alpha M (CD11b)	*Ampd1*	adenosine monophosphate deaminase 1
8	*Efr3b*	EFR3 homolog B	*Itgam*	integrin subunit alpha M (CD11b)
9	*Slc8a2*	solute carrier family 8 member A2	*Slc8a2*	solute carrier family 8 member A2
10	*Matn4*	matrilin 4	*Gdf9*	growth differentiation factor 9
11	*Cxcl2*	C-X-C motif chemokine ligand 2	*Matn4*	matrilin 4
12	*Gdf9*	growth differentiation factor 9	*Mc1r*	melanocortin 1 receptor
13	*Mc1r*	melanocortin 1 receptor	*Cxcl2*	C-X-C motif chemokine ligand 2
14	*Edn1*	endothelin 1	*Edn1*	endothelin 1
15	*Egr2*	early growth response 2	*Crabp2*	cellular retinoic acid binding protein 2
16	*Aoc3*	amine oxidase copper containing 3	*Egr2*	early growth response 2
17	*Pde4b*	phosphodiesterase 4B	*Prkcg*	protein kinase C gamma
18	*Crabp2*	cellular retinoic acid binding protein 2	*Aoc3*	amine oxidase copper containing 3
19	*Pygm*	muscle glycogen phosphorylase	*Pde4b*	phosphodiesterase 4B
20	*Prkcg*	protein kinase C gamma	*Pygm*	muscle glycogen phosphorylase
21	*Hspa1a*	heat shock protein family A member 1A	*Hspa1a*	heat shock protein family A member 1A

Table S2. Antibodies used in COMET multiplex immunofluorescence staining.

Marker	Vendor	Catalog	Clone
**B220**	Thermofisher	14-0452-82	RA3-6B2
**CD8**	Thermofisher	14-0195-82	4SM16
**GzmB**	CST	43701	D2H2F
**CD4**	Thermofisher	14-9766-82	4SM95
**FoxP3**	Thermofisher	14-5773-82	FJK-16S
**CD11c**	CST	39143	D1V9Y
**NK1.1**	Thermofisher	MA1-70100	PK136
**CD11b**	ProteinTech	21851-1-Ap	Polyclonal
**F4/80**	CST	25514	D2S9R
**CD86**	CST	20018SF	E5W6H
**CD163**	Thermofisher	MA5-11458	10D6
**PanCK**	CST	171171SF	C11
**CD31**	BD	752574	MEC 13.3
**αSMA**	Abcam	ab5694	Polyclonal
**Collagen1**	Abcam	ab34710	Polyclonal
**FAP**	Abcam	ab207178	EPR20021

Figure S1

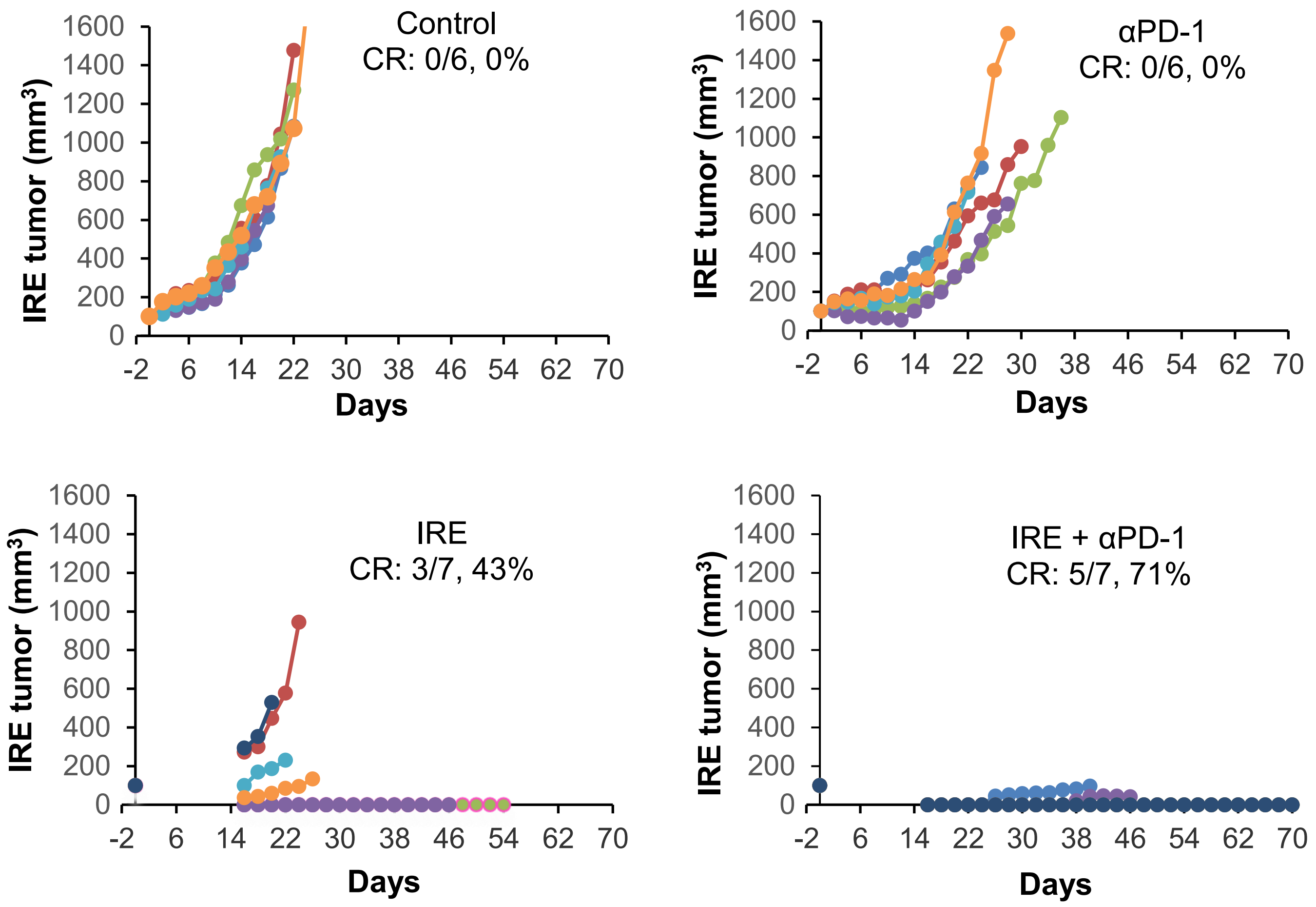

Figure S1. Tumor growth curves for IRE tumors in mice inoculated with KRAS* tumors in both flanks subcutaneously (n=6-7). CR, complete response.

Figure S2

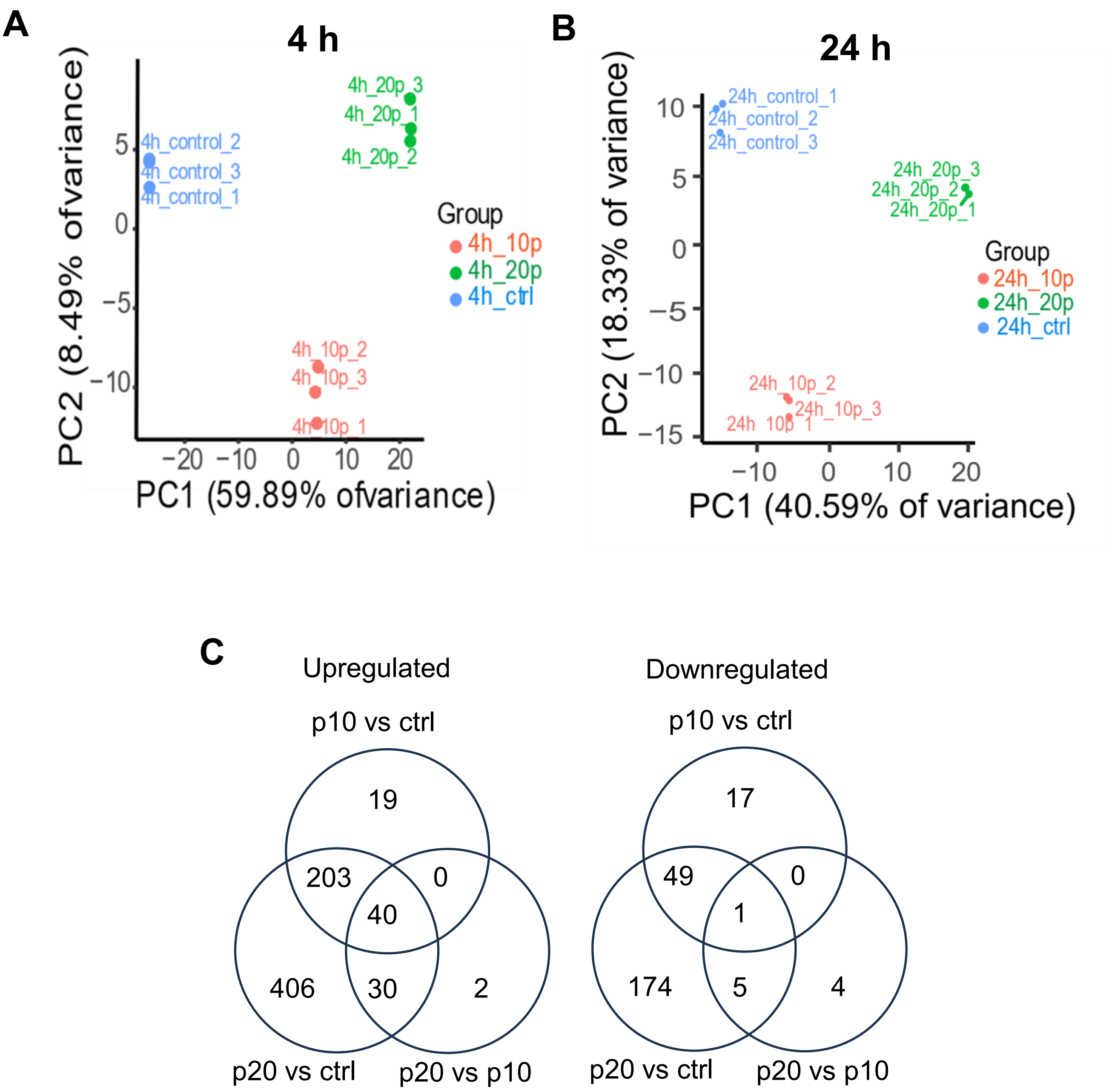

Figure S2. RNA-seq analysis of KRAS* cells at 4 h and 24 h after IRE treatment. (**A, B**) Principal component analysis showing gene expression profiles for KRAS* cells at 4 h (**A**) and 24 h (**B**) after IRE (400 V). The plot demonstrates a clear separation between the three experimental conditions based on their gene expression similarities. Control, no treatment; 10p, IRE with 10 pulses; 20p, IRE with 20 pulses. (**C**) Venn diagram showing the numbers of DEGs with $|log_2FC| > 2.0$ and false discovery rate < 0.01 in comparisons of different sets of data.

Figure S3

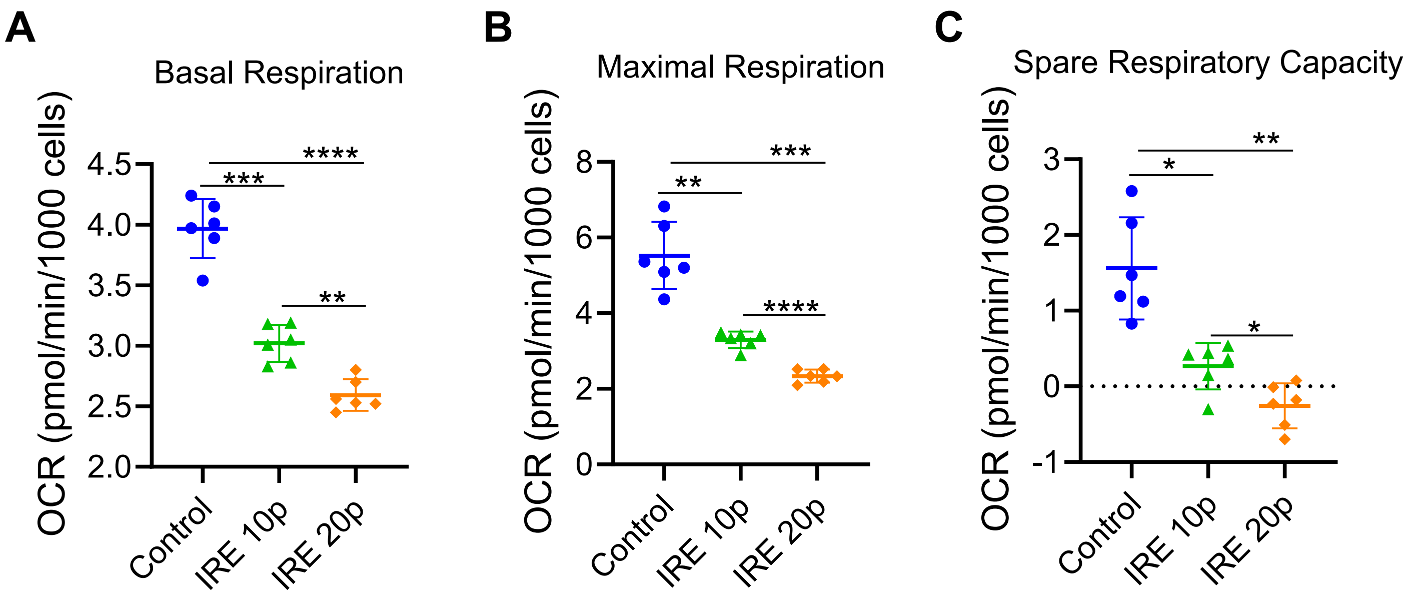

Figure S3. IRE induced mitochondrial dysfunction. Effect of IRE on basal respiration (**A**), maximal respiration (**B**), and spare respiratory capacity (**C**). KRAS* cells were analyzed at 4 h after 10p IRE or 20p IRE (400 V). Data are normalized to cell number at the end of the assay (pmol/min/1000 cells) and are expressed as mean ± SD (n = 6/group). OCR, oxygen consumption rate; ECAR, extracellular acidification rate. *p<0.05, **p<0.01, ***p<0.001, ****p<0.0001 (one-way ANOVA).

Figure S4

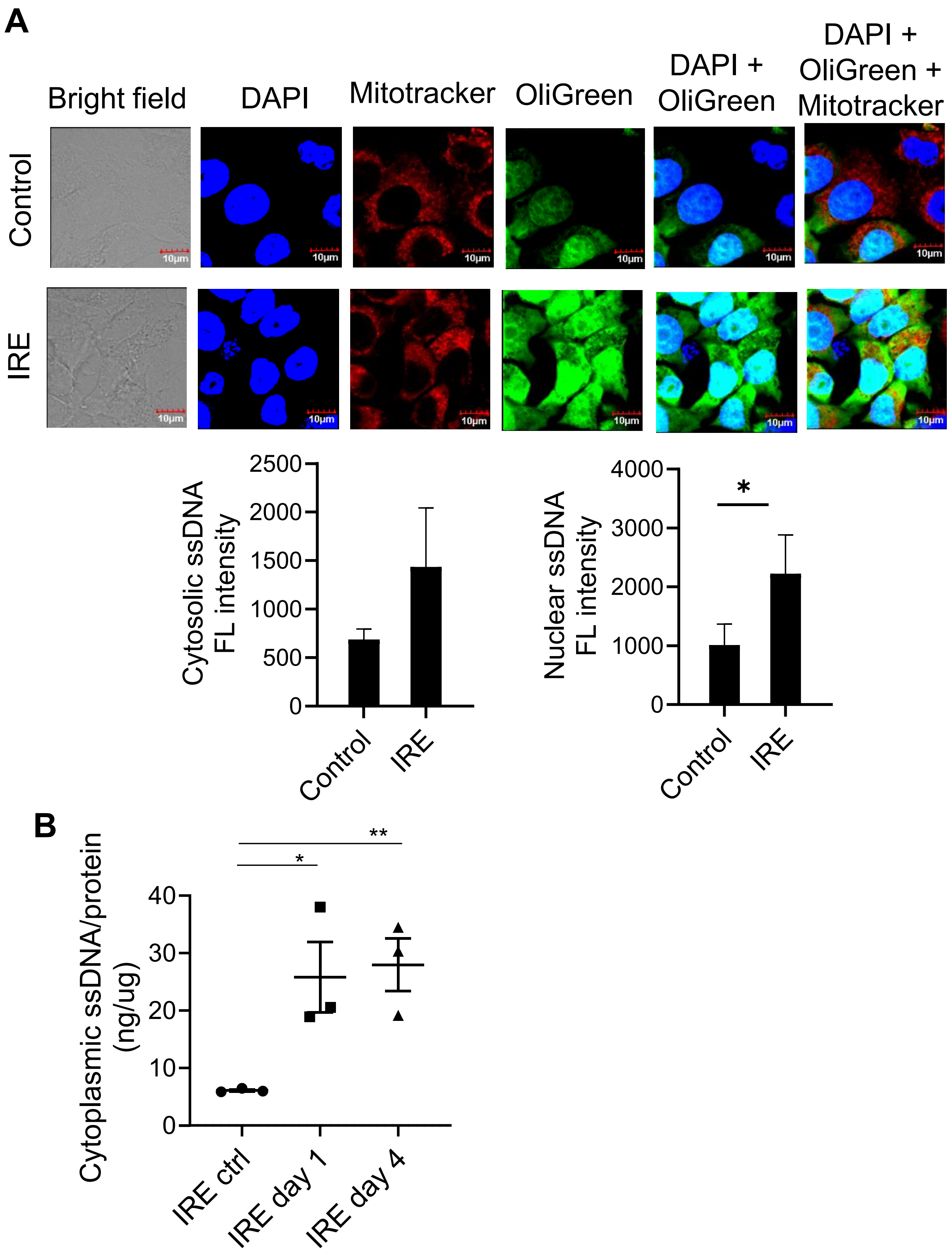

Figure S4. IRE induced cytosolic release of ssDNA. (**A**) Representative confocal fluorescence microscopic images of cytoplasmic ssDNA and corresponding quantification of fluorescence (FL) signal intensity in cytosol and nuclei. PANC-1 cells were stained with OliGreen for ssDNA 24 h after IRE (400 V, 20 pulses). Untreated cells were used as a control. Data are expressed as mean ± SD (n=4). *p<0.05 (Student's t test). (**B**) Analysis of ssDNA from IRE-treated KRAS* tumors using the Qubit ssDNA assay kit. KRAS* tumor tissues were collected on day 1 and day 4 after IRE (1200 V, 99 pulses) and processed for ssDNA assays. *p<0.05, **p<0.01 (Student's t test).

Figure S5

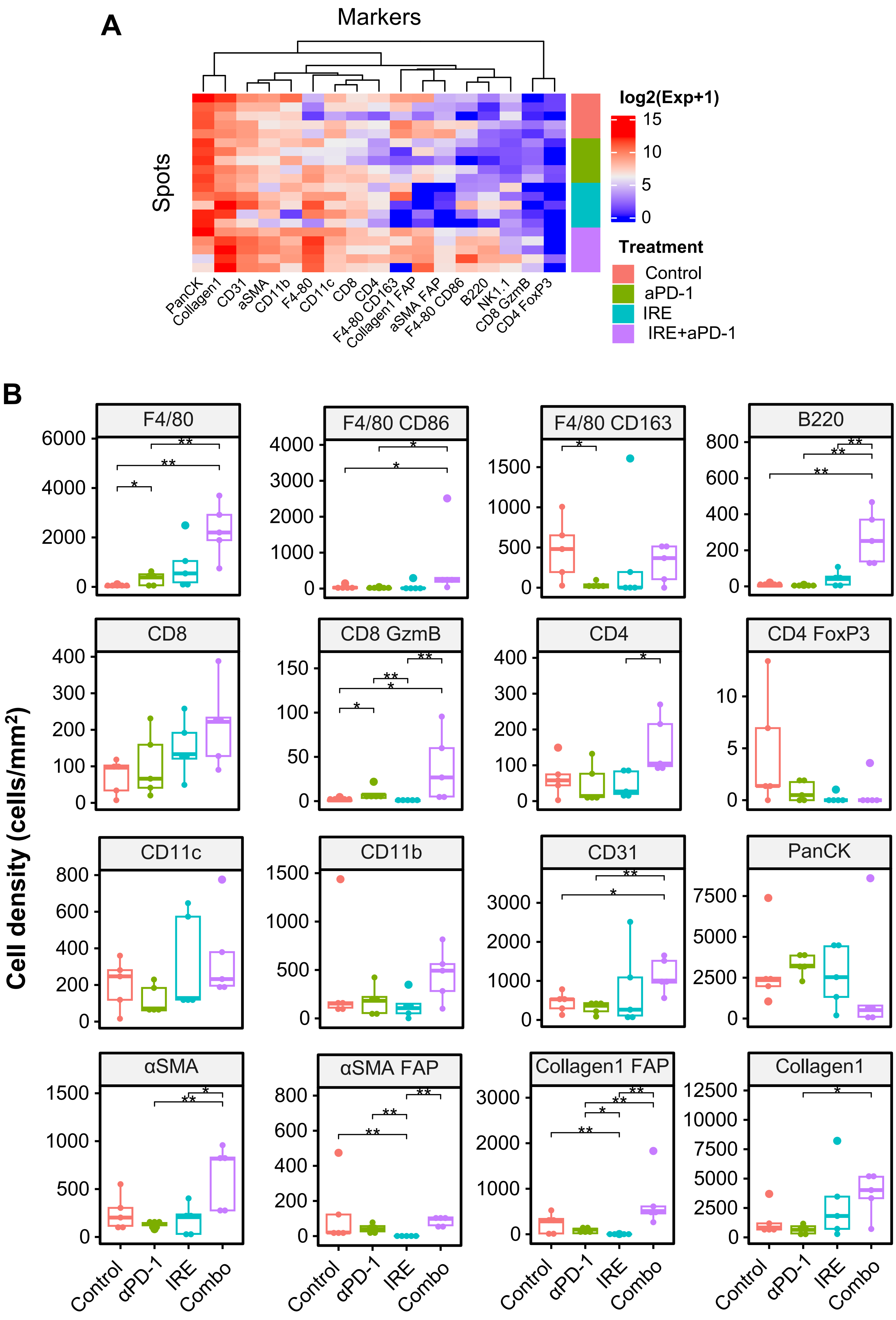
A
Markers
Spots
PanCK
Collagen1
CD31
aSMA
CD11b
F4-80
CD11c
CD8
CD4
F4-80 CD163
Collagen1 FAP
aSMA FAP
F4-80 CD86
B220
NK1.1
CD8 GzmB
CD4 FoxP3
log2(Exp+1)
15
10
5
0
Treatment
Control
aPD-1
IRE
IRE+aPD-1
B
Cell density (cells/mm²)
F4/80
F4/80 CD86
F4/80 CD163
B220
CD8
CD8 GzmB
CD4
CD4 FoxP3
CD11c
CD11b
CD31
PanCK
αSMA
αSMA FAP
Collagen1 FAP
Collagen1
Control
αPD-1
IRE
Combo

Figure S5. IRE + αPD-1 remodeled the TME to turn immunologically "cold" orthotopic KRAS* PDAC tumors into "hot" tumors. (**A**) Heatmap of proteomic biomarkers from P3 and P4 spots of TMA slides after indicated treatments. Data are expressed as $\log_2$(Exp+1). (**B**) Quantitative analysis of cellular biomarkers from multiplex IF images. Cell densities are expressed as mean ± SD (n=3-5/treatment x 4 spots/tumor). *p<0.05, **p<0.01 (two-sample Wilcoxon test). Experimental setup and treatment conditions were the same as in Figure 4A.

Figure S6

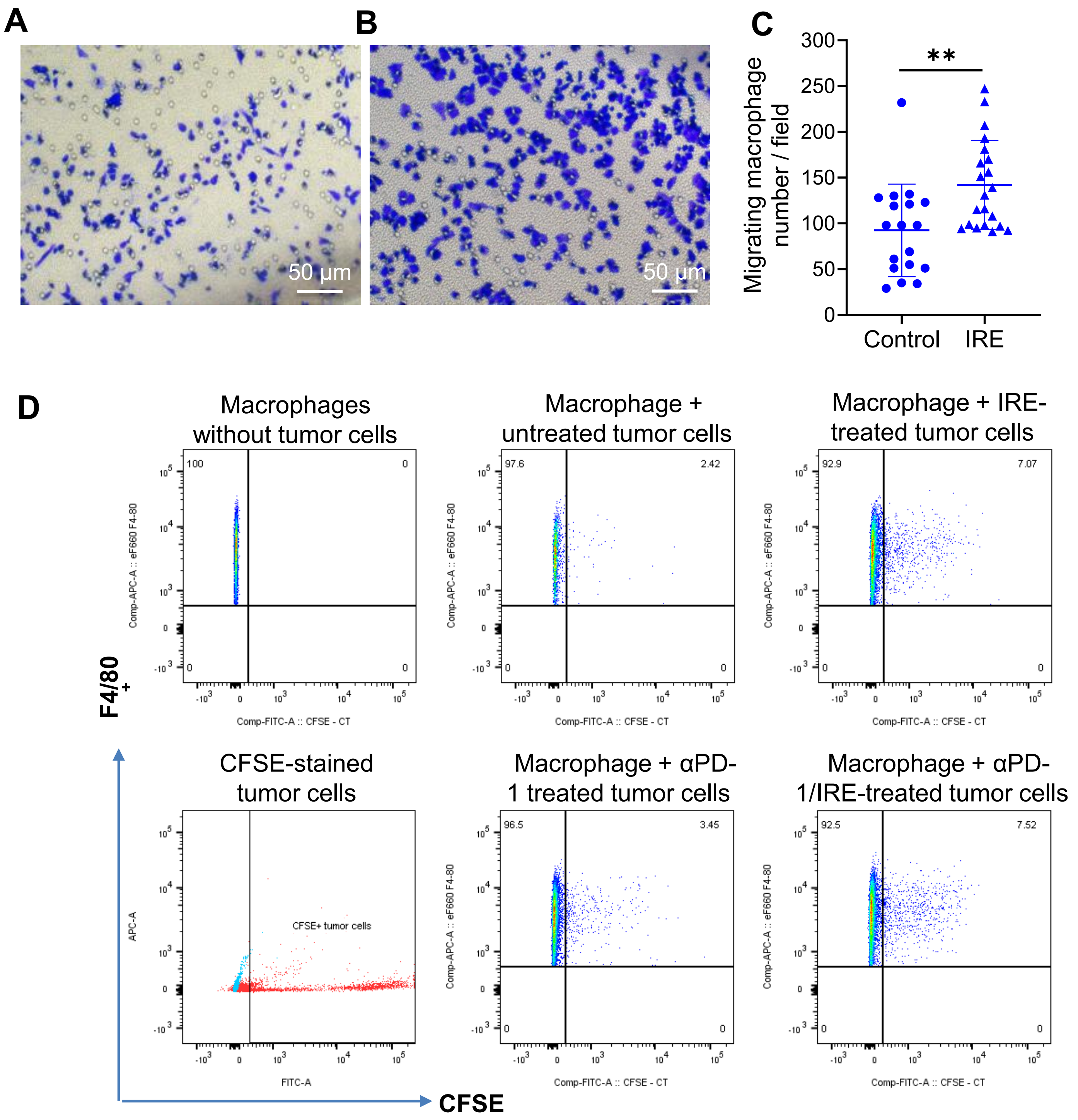

Figure S6. IRE-treated KRAS* cells enhanced macrophage migration and phagocytic activity. (**A, B**) Microphotographs of RAW264.7 macrophages in the upper chamber of a Transwell plate. Cells were co-cultured with untreated tumor cells (**A**) or IRE-treated tumor cells (400 V, 20 pulses) (**B**) seeded in the lower chamber of the Transwell plate. Images were taken at 6 h after co-culture. (**C**) Quantitative analysis of number of migrated macrophages per field of view. Data are presented as mean ± SD (n=18-21). ** p<0.01 (Student's t test). (**D**) Flow cytometry dot plots of RAW264.7 macrophages co-cultured with IRE-untreated or IRE-treated (400 V, 10 pulses) murine HY19636 PDAC cells. IRE enhanced the phagocytotic activity of macrophages, as shown by higher number of cells in the dual-positive quadrant indicative of F4/80-stained macrophages and CFSE-labeled tumor cells.

Figure S7

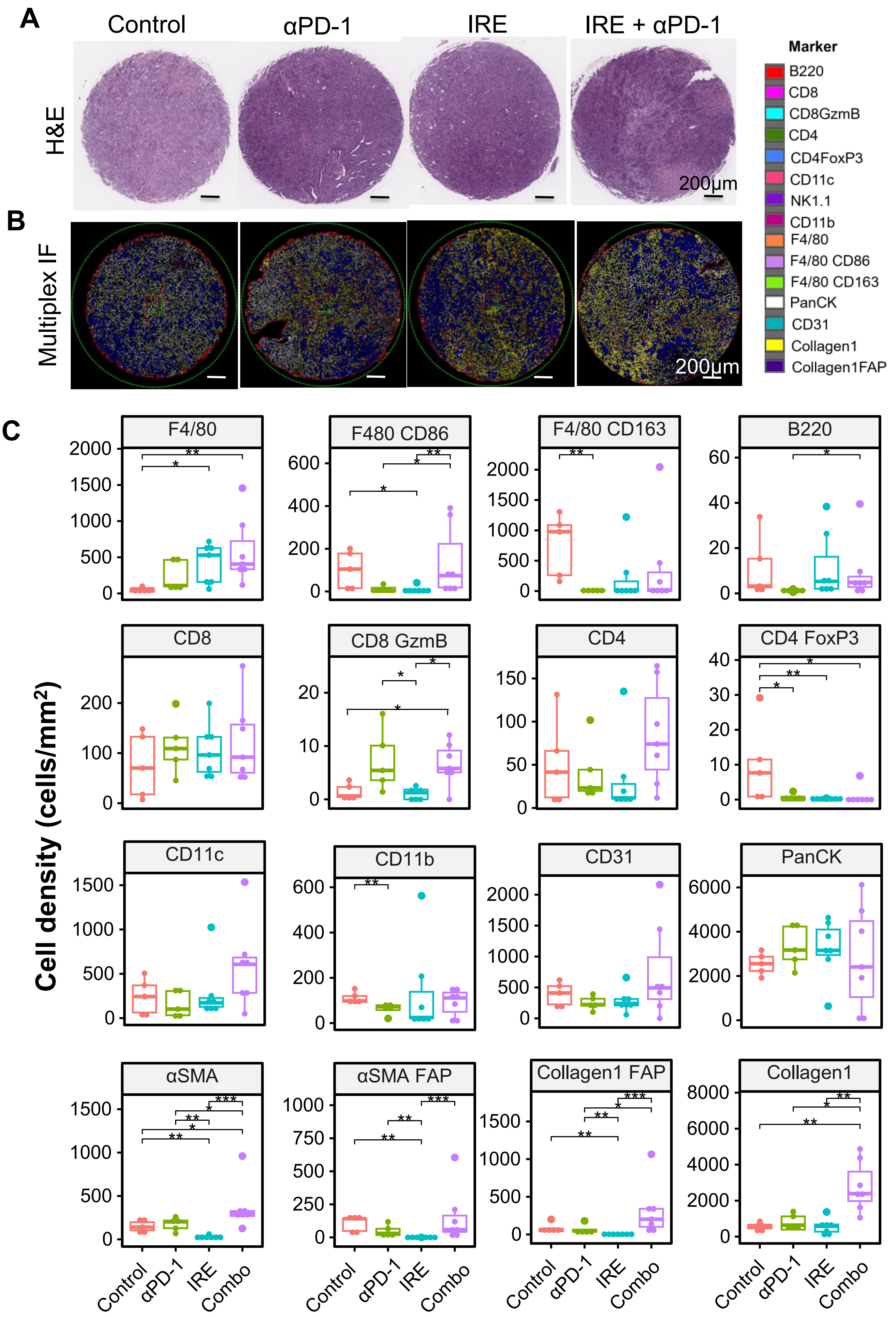
A
Control
αPD-1
IRE
IRE + αPD-1
H&E
200μm
B
Multiplex IF
200μm
Marker
B220
CD8
CD8GzmB
CD4
CD4FoxP3
CD11c
NK1.1
CD11b
F4/80
F4/80 CD86
F4/80 CD163
PanCK
CD31
Collagen1
Collagen1FAP
C
Cell density (cells/mm²)
F4/80
F480 CD86
F4/80 CD163
B220
CD8
CD8 GzmB
CD4
CD4 FoxP3
CD11c
CD11b
CD31
PanCK
αSMA
αSMA FAP
Collagen1 FAP
Collagen1
Control
αPD-1
IRE
Combo

Figure S7. Multiplex IF images of zones P1 and P2 of KRAS* tumors from pancreas and corresponding data analysis. (**A, B**) Representative H&E-stained TMA slides (**A**) and corresponding multiplex IF images (**B**) of zones P1 and P2 of tumors from each of four treatment groups. (**C**) Quantification of different phenotypes of cells in zones P1 and P2. Cell densities are expressed as mean ± SD (n=3-5 tumors/group x 4 spots/tumor). *$p<0.05$, **$p<0.01$, ***$p<0.001$ (two-sample Wilcoxon test). Experimental setup and treatment conditions were the same as in Figure 4A.

Figure S8

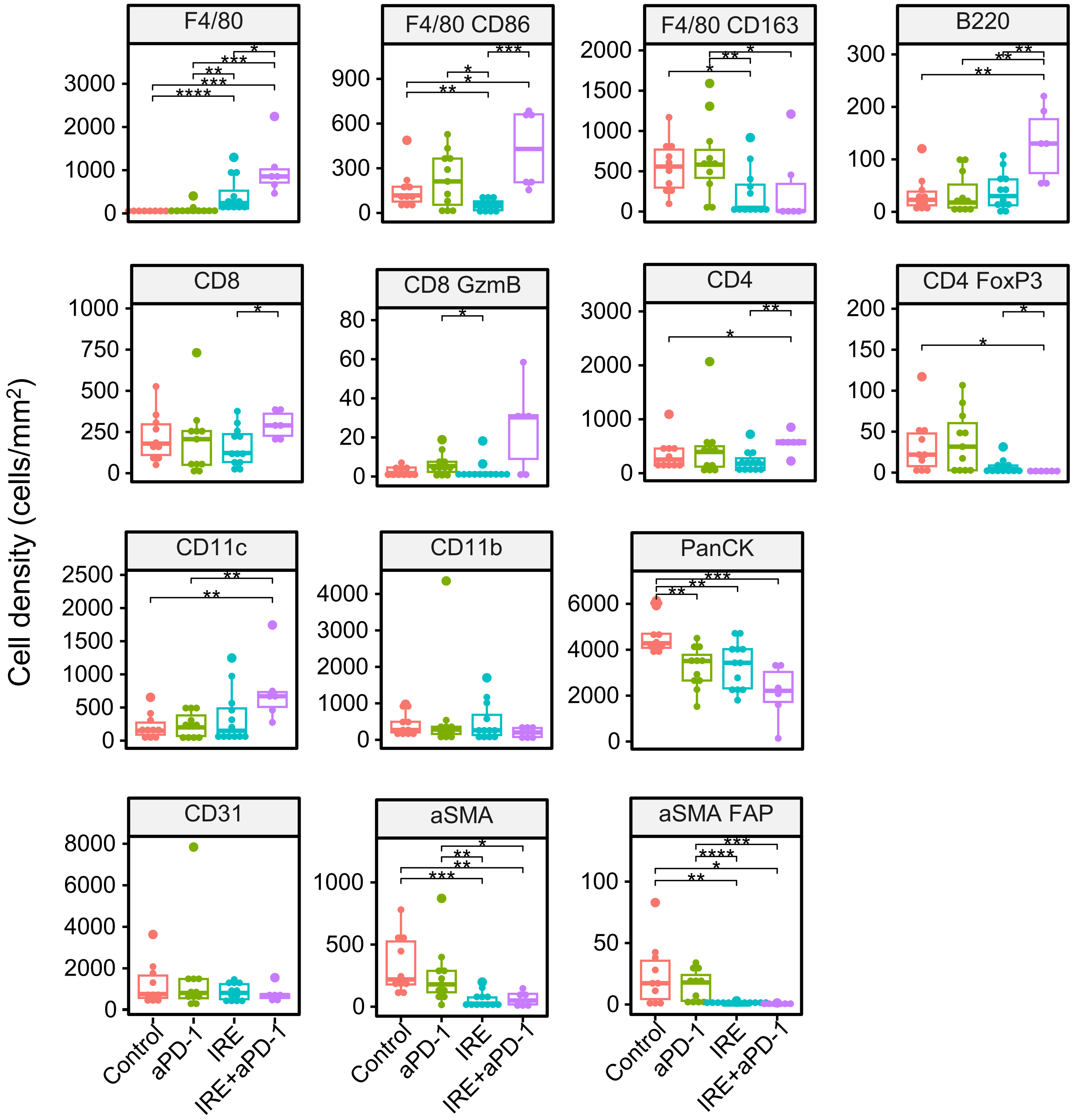

Figure S8. IRE + αPD-1 significantly impacted the TME of abscopal liver metastases. (Quantification of different immune cells in the TME. Cell densities (cells/mm^2) are expressed as mean ± SD (n=3-5 tumors/group × 4 spots/tumor). *p<0.05, **p<0.01, ***p<0.001, ****p<0.0001 (two-sample Wilcoxon test). Experimental setup and treatment conditions were the same as in Figure 4A.

Figure S9

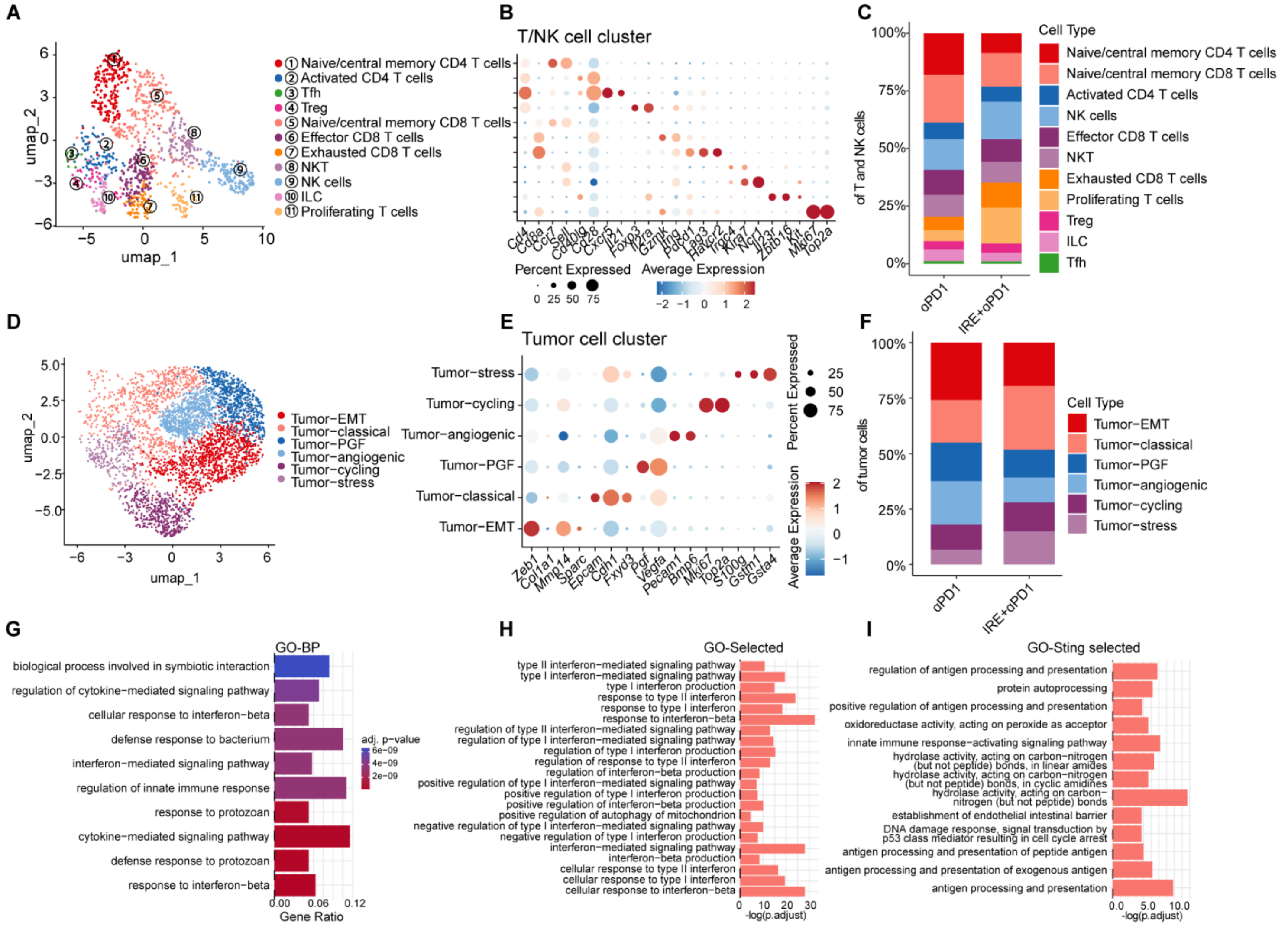

Figure S9. scRNA-seq of orthotopic KRAS* pancreatic tumors on day 5 after initiation of IRE + αPD-1 or αPD-1 alone. (**A-C**) Results of further analysis of the T/NK cell cluster. (**A**) UMAP plot of subclusters. (**B**) Dot plot showing the representative markers for each cluster. The color represents the expression level; the dot size represents the proportion of cells expressing the marker. (**C**) Stacked bar plot showing the populations (i.e., percentages of all cells) of the T/NK subcluster in the αPD-1 and IRE + αPD-1 groups. There were more than three times as many proliferating T cells in the IRE + αPD-1 group as in the αPD-1 alone group, and most proliferating T cells were CD8a$^+$ T cells. (**D-I**) Results of further analysis of the tumor epithelial cell cluster. (**D**) UMAP plot of subclusters. (**E**) Dot plot showing the representative markers for each cluster. (**F**) Stacked bar plot showing the populations (i.e., percentages of all cells) of the tumor epithelial subcluster in the αPD-1 and IRE + αPD-1 groups. (**G**) Bar plots showing the functional enrichment of DEGs of tumor epithelial cells in GO biological processes in the IRE + αPD-1 group (gene ratio > 0) versus the αPD-1 alone group (gene ratio < 0). Bar plots showing the selected GO pathways of interferon-related (**H**) and STING-related (**I**) processes of genes upregulated in the IRE + αPD-1 group versus the αPD-1 alone group.

Figure S10

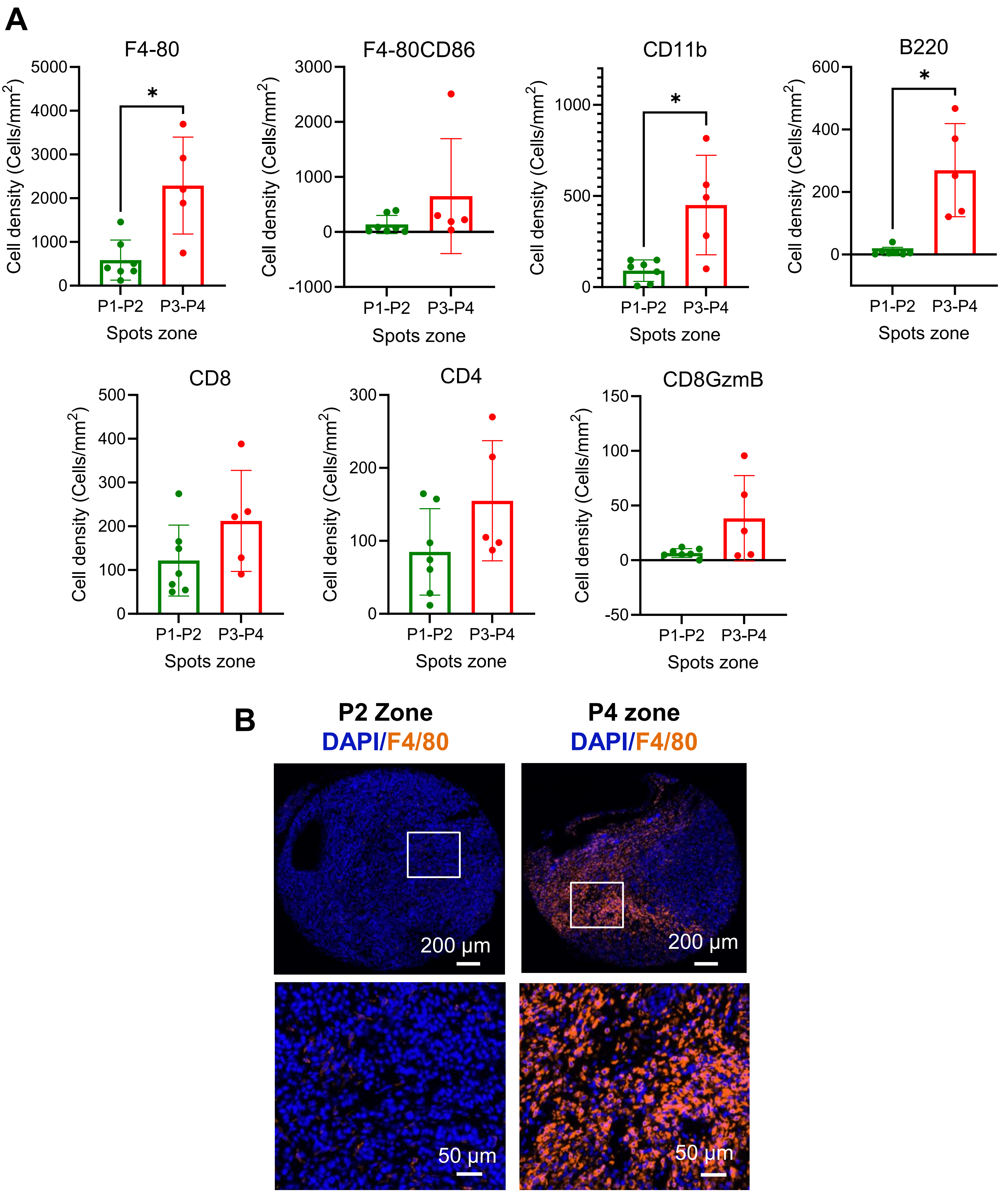

Figure S10. Analysis of spatial proteomic profiling of selected immune cells in orthotopic KRAS* PDAC tumors treated with IRE + αPD-1. (A) Cell density of selected immune cells comparing Zone P1-P2 versus P3-P4. (B) Representative images of tumor-associated macrophages in zone P2 versus zone P4.
